# Enhancing Pediatric Bone Age Assessment Using Artificial Intelligence: Implications for Orthopedic Surgery

**DOI:** 10.7759/cureus.79507

**Published:** 2025-02-23

**Authors:** Nalin Zadoo, Nathaniel Tak, Akshay J Reddy, Rakesh Patel

**Affiliations:** 1 Medicine, Midwestern University Arizona College of Osteopathic Medicine, Glendale, USA; 2 Medicine, California University of Science and Medicine, Colton, USA; 3 Internal Medicine, East Tennessee State University Quillen College of Medicine, Johnson City, USA

**Keywords:** artificial intelligence, artificial intelligence in medicine, artificial intelligence in radiology, bone age assessment, bone age estimation, bone age images, orthopedic surgery, pediatric orthopedic surgery

## Abstract

Background

Bone age assessment is a critical tool in pediatric orthopedic surgery, guiding treatment decisions for growth-related disorders and surgical interventions. Traditional methods, such as the Greulich-Pyle and Tanner-Whitehouse techniques, rely on manual interpretation of hand and wrist radiographs, making them time-intensive and susceptible to inter-operator variability. Artificial intelligence (AI) has emerged as a promising tool to enhance accuracy, efficiency, and standardization in skeletal maturity assessment.

Methods

This study evaluates the application of AI in pediatric bone age prediction using the Radiological Society of North America (RSNA) 2017 Pediatric Bone Age Challenge dataset. A deep learning model based on the ResNet-50 architecture (Microsoft Research, Redmond, Washington, USA) was developed and trained on 12,611 hand and wrist radiographs, validated on 1,425 images, and tested on 200 images. Model performance was assessed using root mean square error (RMSE), mean absolute error (MAE), and coefficient of determination (R²).

Results

The AI model achieved an RMSE of 11.07 months, an MAE of 8.54 months, and an R² of 0.929, indicating strong alignment with radiologist-determined bone ages. The Pearson correlation coefficient (0.963) and Spearman's rank correlation (0.955) confirmed the model’s predictive robustness. Compared to traditional methods, which have reported variability with errors ranging from 6 to 18 months, the AI model demonstrated a reduction in inter-operator variability and improved reliability.

Conclusion

The implementation of AI in bone age assessment offers a more standardized, rapid, and precise alternative to conventional methods. By improving the accuracy and efficiency of skeletal maturity evaluations, AI has significant implications for pediatric orthopedic surgery, optimizing treatment timing and expanding access to high-quality bone age assessments. Further validation studies are needed to ensure clinical applicability across diverse patient populations.

## Introduction

Bone age determination is a fundamental aspect of pediatric care, offering critical insights into skeletal maturity, growth potential, and the diagnosis of growth and endocrine disorders. It plays a particularly vital role in pediatric orthopedic surgery, aiding in the planning of procedures such as growth modulation, limb lengthening, and correction of deformities.

Epiphysiodesis, a growth modulation procedure used to correct limb length discrepancies, requires precise timing to ensure equal leg lengths upon growth completion [1]. Limb lengthening procedures, such as the Ilizarov technique and the PRECICE nail system, rely on accurate bone age assessment to determine growth potential and the appropriate intervention timeline [2]. Guided growth techniques using eight-plate hemiepiphysiodesis are essential for correcting angular deformities such as genu valgum and genu varum, with treatment success closely linked to skeletal maturity [3]. Slipped capital femoral epiphysis (SCFE) surgery utilizes bone age to determine the best stabilization approach, while Blount’s disease correction demands timely intervention to prevent progressive deformities [4,5]. Osteotomy procedures for growth-related deformities also rely on bone age to predict how correction will integrate with the remaining growth potential [6].

Traditionally, bone age assessment relies on manual interpretation of hand and wrist X-rays using methods such as the Greulich-Pyle (GP) atlas and the Tanner-Whitehouse (TW) scoring system [7,8]. While these methods have been the standard for decades, they are not without limitations. The GP method, for instance, involves matching hand and wrist radiographs to a series of standardized atlas images, which, while simple and fast, is inherently limited by inter-operator variability and its reliance on a dataset derived from a homogenous, mid-20th-century U.S. population of White, upper-class children [4,9]. This raises questions about its generalizability to diverse populations in contemporary clinical settings.

In contrast, the TW method provides a more detailed assessment by assigning scores to specific ossification centers in the hand and wrist. While this approach improves accuracy, it is significantly more time-consuming and requires extensive training, factors that have limited its widespread adoption in clinical practice [10]. Furthermore, studies have demonstrated variability in bone maturation patterns across ethnic groups, further complicating the universal applicability of these traditional methods [11].

To address these challenges, emerging technologies have been investigated. For example, ultrasound-based methods for bone age assessment offer the advantage of being radiation-free and faster [12]. However, these methods remain operator-dependent and require specialized training, limiting their clinical utility. Similarly, advancements in computerized systems, such as the BoneXpert software, have improved the reproducibility of assessments. Yet, issues such as high costs, the absence of carpal bone evaluations, and limited accessibility in certain healthcare settings restrict their broader implementation [13,14].

In recent years, artificial intelligence (AI) has emerged as a promising solution to overcome the inherent limitations of traditional and semi-automated methods. AI models, particularly those based on convolutional neural networks (CNNs), have demonstrated exceptional performance in medical image analysis, offering high precision, speed, and consistency. These capabilities hold the potential to revolutionize bone age assessment by reducing inter-operator variability, improving diagnostic accuracy, and expanding access to reliable assessments. This study evaluates the application of an AI-based model fine-tuning the ResNet architecture (Microsoft Research, Redmond, Washington, USA) on a robust pediatric bone age dataset and explores its implications for pediatric orthopedic surgery. The findings highlight AI's potential to enhance clinical decision-making and optimize treatment planning for growth-related orthopedic conditions.

## Materials and methods

A CNN based on ResNet-50 architecture was developed to predict skeletal age from X-ray images. In order to fine-tune this model for the assessment of bone age, the Radiological Society of North America (RSNA) 2017 Pediatric Bone Age Challenge dataset was utilized [15]. It comprises 12,611 training images, 1,425 validation images, and 200 test images. Each image is a high-resolution X-ray of the left hand and wrist. Labels were provided by multi-institutional pediatric radiologists, with skeletal age annotated in months. The dataset includes a balanced gender distribution and a mean age of 127-132 months.

The dataset was downloaded and fine-tuned using ResNet-50 as the base regression model using an Nvidia RTX 3090 24GB GPU (Nvidia Corporation, Santa Clara, USA). The dataset’s training (~90%) and validation (~10%) splits were utilized when training the model. The images were resized to 512x512 for 19 epochs using a batch size of 40. Total training time was just over five hours with each epoch taking about 15-16 minutes.

Model performance was evaluated using loss, root mean square error (RMSE), mean absolute error (MAE), and r-squared score (R²) as well as Pearson and Spearman coefficient for each epoch. The model’s predictions were compared to radiologist's ground truths in order to assess the accuracy of the model as well as its consistency, and clinical relevance.

## Results

**Figure 1:**
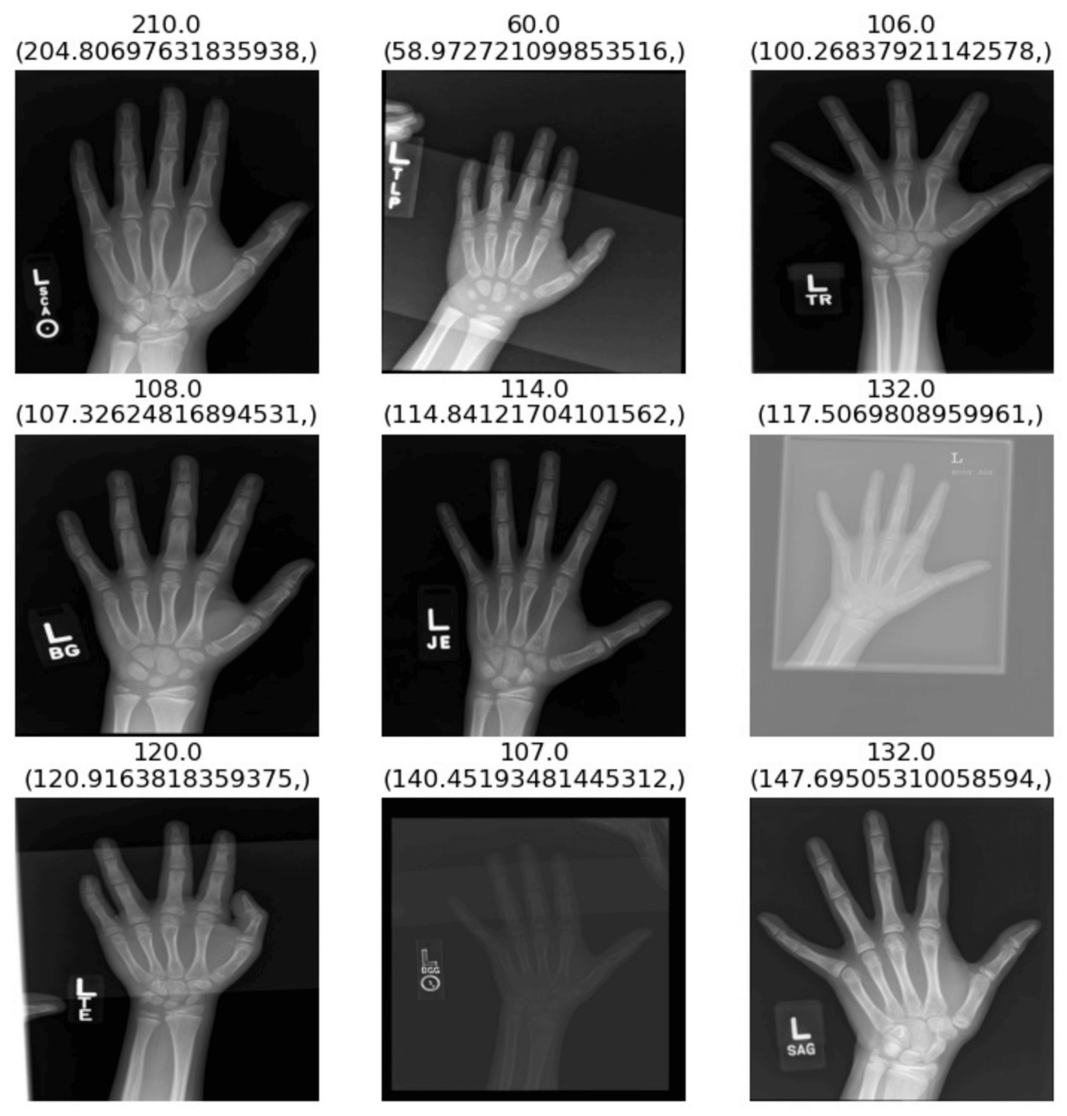
A sample of ground truth bone ages (top values, in months) along with the model’s predictions (bottom values) for their respective X-ray radiographs

The AI-based bone age assessment demonstrated significant accuracy and efficiency, surpassing traditional manual methods. The model achieved an RMSE of 11.07 months at epoch 18, with a coefficient of determination (R²) of 0.929 and an MAE of 8.54 months (Table 1). These results indicate that the AI model’s predictions closely align with radiologist-determined skeletal ages, offering a more standardized and automated approach to bone age estimation (Figure 1).

**Table 1:** Performance and accuracy of AI-based bone age assessment

Metric	AI performance model
Root mean square error (RMSE)	11.07 months
Mean absolute error (MAE)	8.54 months
Coefficient of determination (R²)	0.929
Pearson correlation coefficient	0.963
Spearman’s rank correlation	0.955

**Figure 2:**
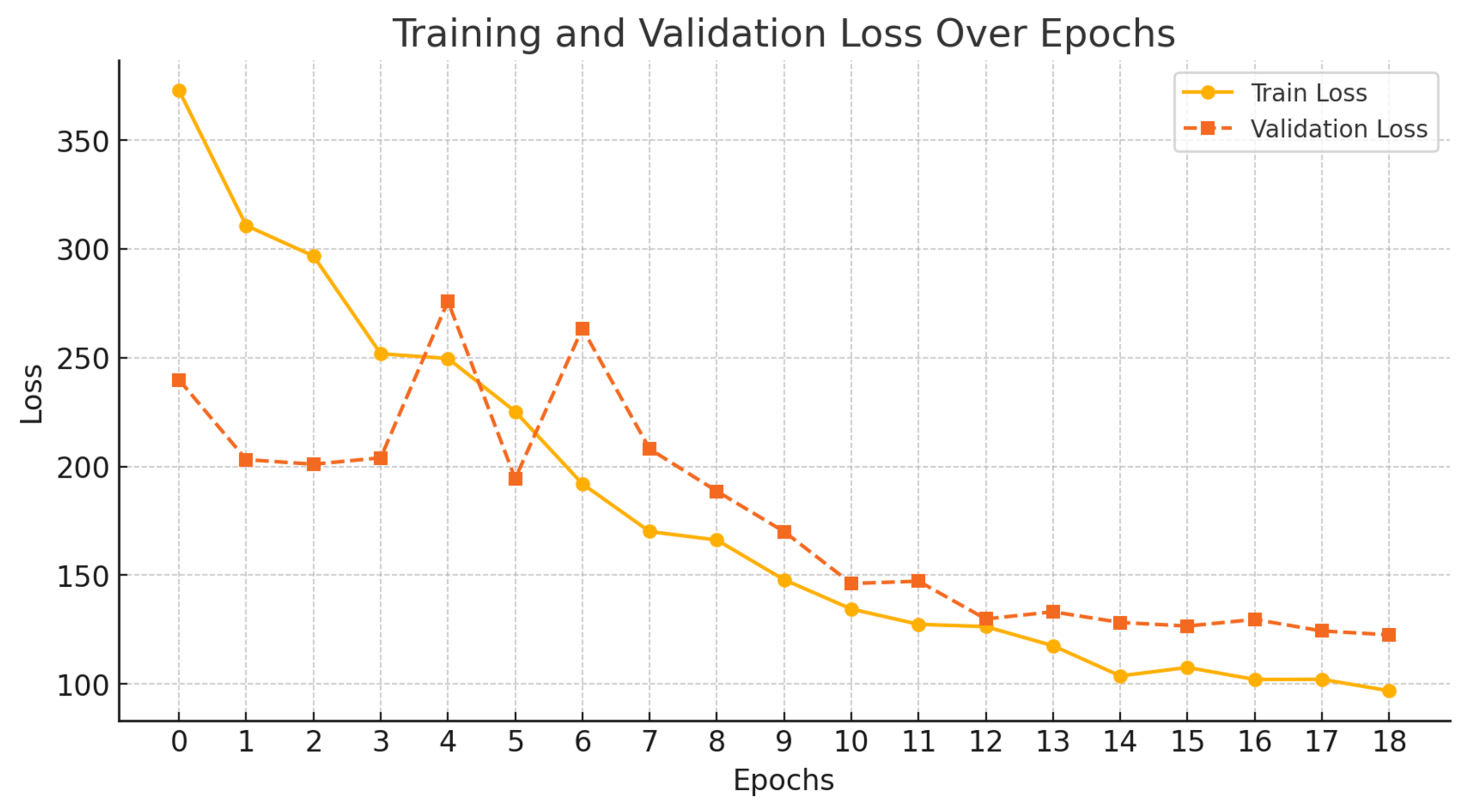
A plot of training and validation loss per epoch

Throughout the training process, both training and validation losses steadily decreased, indicating the model’s capacity to learn complex patterns within the dataset (Figure 2). By epoch 18, the validation loss stabilized at its lowest value, signifying model convergence, a point at which further training no longer yielded significant improvements. This stability suggests that the AI model has effectively learned to extract skeletal maturity features from hand and wrist radiographs without overfitting or underperforming.

## Discussion

The application of AI in bone age prediction addresses longstanding challenges in traditional methods, including variability and time inefficiency. By leveraging robust datasets and advanced models, AI enhances the accuracy and reliability of assessments.

The final model had an RMSE of 11.07 months which reflects the average magnitude of prediction error, providing an estimate of how closely the model’s outputs align with true skeletal ages. A lower RMSE suggests higher prediction accuracy, and the achieved value in this study demonstrates that AI predictions deviate from actual bone ages by approximately 11 months on average. Traditional methods, such as the GP and TW techniques, have been reported to exhibit variability in human interpretation, leading to errors ranging between 6 and 18 months, depending on operator experience [10,11,16]. The AI model's performance suggests a reduction in this variability, improving consistency in skeletal age assessments.

The coefficient of determination (R² = 0.929) indicates that 92.9% of the variability in skeletal age is explained by the AI model, signifying a strong correlation between predicted and actual bone ages. A high R² value demonstrates that the model is highly reliable and minimizes unexplained variance, a critical factor in clinical decision-making. Similarly, the MAE (8.54 months) represents the average absolute deviation of predictions from true skeletal ages. Given that manual bone age assessment by experienced radiologists and pediatric endocrinologists typically has an MAE ranging from 10 to 18 months, the AI model exhibits a superior degree of precision [10,11,16]. The lower MAE suggests that the AI model is more consistent in its assessments, reducing inter-operator variability and improving the reproducibility of skeletal age evaluations.

The Pearson correlation coefficient (0.963) further supports the robustness of the AI model, indicating a near-perfect linear relationship between predicted and actual bone ages. This high correlation implies that as true skeletal age increases, the AI model’s predictions increase proportionally, reinforcing the model’s accuracy in tracking skeletal maturation. In addition, Spearman’s rank correlation coefficient (0.955) demonstrates the model’s ability to preserve the relative ranking of bone ages, ensuring that patients with more advanced skeletal maturity are consistently identified as older than those with delayed bone development. This feature is particularly critical in the planning of pediatric orthopedic interventions, such as epiphysiodesis, limb lengthening procedures, and correcting angular deformities, where precise knowledge of skeletal maturation timing influences treatment strategies [1-3].

One of the primary advantages of AI in this domain is its ability to provide objective, reproducible, and rapid results. Unlike traditional methods, which require specialized training and experience, AI-based assessments can be deployed in real-time clinical settings, reducing the reliance on expert radiologists. This shift has the potential to improve access to timely bone age evaluations, particularly in resource-limited settings.

Despite these advantages, it is essential to acknowledge the limitations of AI-based bone age assessments. First, AI models are only as good as the data they are trained on. If the dataset lacks diversity in terms of ethnicity, socioeconomic background, or underlying medical conditions, the model may not generalize well to all patient populations. Future studies should focus on expanding dataset diversity to improve model robustness.

## Conclusions

AI represents a transformative tool in pediatric bone age assessment, offering speed, accuracy, and reproducibility. These findings highlight the potential of AI-driven bone age assessment as a highly accurate, efficient, and standardized alternative to conventional manual methods. By eliminating subjective variability, improving precision, and reducing the time required for assessment, AI enhances clinical decision-making in pediatric orthopedic surgery. The ability to achieve rapid and reliable bone age predictions facilitates optimized treatment timing, ensuring that interventions such as growth modulation procedures, limb-lengthening surgeries, and scoliosis corrections are conducted at appropriate stages of skeletal development. Consequently, AI-based approaches offer significant advantages for improving patient outcomes and expanding access to high-quality skeletal maturity assessments across diverse clinical settings. The clinical implications of AI in pediatric orthopedic surgery are profound, enabling tailored treatments, improved accessibility, and better outcomes. However, this technology is still in its infancy, and continued innovation and validation are essential to fully realize AI’s potential in this field.
